# Métastases septique neuro-méningée suite à une otite à pneumocoque

**DOI:** 10.11604/pamj.2015.20.164.4358

**Published:** 2015-02-23

**Authors:** Coulibaly Mahamadoun, Mohamed Khatouf

**Affiliations:** 1 Service d'Anesthésie-Réanimation Polyvalente A1, CHU Hassan II de Fès, Maroc

**Keywords:** Métastases, méningite à Pneumocoque, Otite purulente, metastases, Pneumococcal meningitis, purulent otitis

## Image en medicine

Nous rapportons l'observation médicale d'un patient de 39 ans admis dans notre formation pour méningite à Pneumocoque à point de départ ORL (Otite purulente) non traité 10 jours auparavant. L'évolution était favorable sous Ceftriaxone 50mg/kg/12h. La TDM trouvait un oedème cérébral diffus compatible avec une méningo-encéphalite. L'imagerie par résonnance magnétique (IRM) trouvait des lésions faisant évoquer une métastase septique.

**Figure 1:**
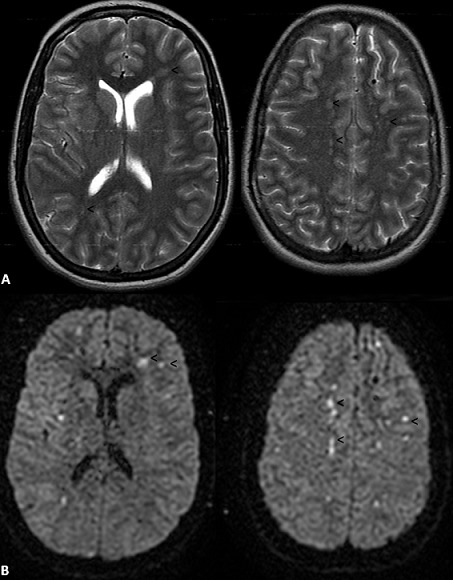
IRM cérébrale: Séquences dans le plan axial en pondération T2 (A) avec images correspondantes en séquence de diffusion (B), objectivant de multiples lésions millimétriques sus-tentorielles en hypersignal T2, associées à une restriction de la diffusion (hyper intensités : tête de flèche) et intéressant des territoires vasculaires multiples compatibles avec une origine embolique

